# Circulating Tumor Cells: What Is in It for the Patient? A Vision towards the Future

**DOI:** 10.3390/cancers6021195

**Published:** 2014-05-28

**Authors:** Anja van de Stolpe, Jaap M. J. den Toonder

**Affiliations:** 1 Fellow, Precision and Decentralized Diagnostics, Philips Research, Eindhoven 5656 AE, The Netherlands; 2 Chair Microsystems, Eindhoven University of Technology, Postbox 513, Eindhoven 5600 MB, The Netherlands; E-Mail: J.M.J.d.Toonder@tue.nl

**Keywords:** cancer, metastasis, circulating tumor cell, CTC, digital pathology, companion diagnostics, cell-free DNA, organ-on-chip, computational pathway model

## Abstract

Knowledge on cellular signal transduction pathways as drivers of cancer growth and metastasis has fuelled development of “targeted therapy” which “targets” aberrant oncogenic signal transduction pathways. These drugs require nearly invariably companion diagnostic tests to identify the tumor-driving pathway and the cause of the abnormal pathway activity in a tumor sample, both for therapy response prediction as well as for monitoring of therapy response and emerging secondary drug resistance. Obtaining sufficient tumor material for this analysis in the metastatic setting is a challenge, and circulating tumor cells (CTCs) may provide an attractive alternative to biopsy on the premise that they can be captured from blood and the companion diagnostic test results are correctly interpreted. We discuss novel companion diagnostic directions, including the challenges, to identify the tumor driving pathway in CTCs, which in combination with a digital pathology platform and algorithms to quantitatively interpret complex CTC diagnostic results may enable optimized therapy response prediction and monitoring. In contrast to CTC-based companion diagnostics, CTC enumeration is envisioned to be largely replaced by cell free tumor DNA measurements in blood for therapy response and recurrence monitoring. The recent emergence of novel *in vitro* human model systems in the form of cancer-on-a-chip may enable elucidation of some of the so far elusive characteristics of CTCs, and is expected to contribute to more efficient CTC capture and CTC-based diagnostics.

## 1. Introduction

Systemic treatment of cancer is expected to change dramatically in the coming years. While conventional chemotherapy treatment kills dividing cells, novel drug-based treatments aim at targeting the abnormal biology underlying cancer growth and metastasis [1]. Since the initial sequencing of the human genome in 2000, knowledge on the multitude of encoded proteins and their cellular functions has rapidly increased. This has had tremendous impact on cancer research. With it came the identification and characterization of cellular signal transduction pathways which drive cancer growth and metastasis. This has fuelled development of a whole new category of “targeted drugs”, which “target” aberrant oncogenic signal transduction pathways. Somewhat simplified, every tumor has its “own” tumor driving signalling pathway, which implies that a specific targeted drug will not benefit every patient. A companion diagnostic test which can reliably identify the tumor-driving pathway (and the underlying defect) in the cancer tissue to be treated will be increasingly required to choose the optimal drug (combination), both in a primary tumor as well as in the metastatic cancer setting. In addition, in view of the increasing number of targeted drugs to choose from to treat a patient with cancer, it will also become high priority to be able to rapidly assess whether the chosen targeted therapy is indeed effective, or whether after a certain treatment time secondary resistance develops. This assessment is important to allow a timely switch to another, potentially more effective, targeted drug or drug combination.

Many currently available companion diagnostic tests lack in desired sensitivity and specificity, example cases are the ER (estrogen receptor) and Her2/Neu pathology staining tests used in patients with breast cancer to decide on hormonal and trastuzumab treatment respectively. In addition, in metastatic patients treatment choice is nearly invariably based on performing such tests on the primary tumor tissue, instead of tissue of the metastatic tumors that need to be treated, and of which we know that their biology, including the tumor driving pathway, is often different from the primary tumor [2,3,4,5].

Two main clinical questions can be formulated for optimizing treatment of metastasized cancer patients: (1) how can companion diagnostic tests be improved to identify with high sensitivity and specificity the tumor driving pathway including the underlying cellular or genomic defect; and (2) how can tumor material be obtained that is sufficiently representative for the metastases to be treated, and on which these companion tests can be adequately performed. These questions need to be answered to enable the clinical oncologist to choose the optimal targeted drug therapy for the right patient. If new treatment options like immunotherapy will join the cancer therapy arena, pathway-targeted therapies are expected to remain a cornerstone of systemic cancer treatment.

In this conceptual paper we will address these two questions in the light of a few highly interesting recent technical developments, that, when combined in a right way, are envisioned to provide powerful solutions: (a) novel approaches to capture circulating tumor cells (CTCs) from blood; (b) novel assays to assess signal transduction pathway activity in a tissue or cell sample; (c) digital scanner-software combinations enabling digitization of pathology and cytology (including CTCs) slides; and finally (d) the emergence of multiplex fluorescent pathology staining techniques in combination with development of powerful algorithms to interpret the resulting complex data.

## 2. The Problem of Companion Diagnostic Testing: Cancer Genotype *versus* Phenotype

Roughly ten different major signal transduction pathways can drive tumor growth: estrogen and androgen receptor pathways (ER, AR), the developmental pathways Wnt, Notch, Hedgehog, TGFbeta, and FGF, the inflammatory NFkappaB pathway, and a number of related growth factor PI3K pathways [6,7,8,9,10,11,12,13,14]. With the increasing availability of targeted drugs, each directed towards blocking one of these pathways, for every cancer the responsible pathway needs to be identified in order to choose the right drug (combination). The respective tumor driving signalling pathway can differ between tumors of different cellular origin, but also between primary tumors of the same kind, like for example ER positive breast cancers, between similar tumors in different stages of progression, like a primary *versus* a metastatic tumor, and even within one and the same tumor due to the presence of multiple cancer cell clones or differences in the microenvironment of the tumor cells; finally, within metastatic tumors different pathways may conceivably become activated depending on organ location and associated locally defined conditions [15,16]. The influence of the microenvironment on cancer cell biology and pathway activity is by now firmly established and implies that determining signalling pathway activity in a cancer sample based solely on mutation information obtained from the sequenced genome or exome of the cancer cell is often confounding, and may lead to erratic results [17]. Recently a novel approach to develop diagnostic probabilistic computational models to assess “phenotypic” signalling pathway activity in an individual cancer tissue sample has been described and partially clinically validated [18]. These knowledge-based Bayesian network-type computational models incorporate experimental evidence on pathway-specific target genes to provide a probability of signalling pathway activation, based on a quantitative RNA profile as data-input. In addition, intelligent novel multiplex fluorescent tissue staining technologies are being developed, which may enable assessment of activity status of a signal transduction pathway on a tissue or cytology slide in combination with other cell characterization markers, thus providing important “phenotypic” cancer cell information *in situ* on a single cell basis [19,20,21]. For example an active membrane receptor dimer or a phosphorylated signaling protein within a specific pathway can be stained and discriminated from the non-active form. Combining computational model-based mRNA pathway analysis with such innovative cell stainings makes it possible to identify the type of cell in a tissue or cytology sample in which a specific signal transduction pathway is active. Using such phenotypic information on pathway activity is also expected to guide and facilitate the search for identification of a tumor driving genomic mutation. Whole genome sequencing is increasingly used to identify tumor-driving DNA aberrations in cancer, however due to the large number of “passenger” mutations this has proven to be extremely challenging. Prior knowledge on which signaling pathway is active may make the search for a driving mutation more focused and straightforward.

## 3. Applying Companion Diagnostics in a Metastatic Setting: How to Proceed?

A number of clinical trials have shown that the presence of signaling pathway biomarkers, like for example ER (estrogen receptor) and Her2/Neu in breast cancer, may differ between primary and metastatic tumors, in up to about half of the cases [5]. As a consequence many patients are likely to be treated with drugs that are not effective and in addition have significant side effects—aside from the cost aspect. In view of the ongoing trend to treat cancer more and more with targeted drugs, differences in the tumor driving pathway between the primary tumor and metastatic tumors is emerging as an important clinical problem, since targeted therapy choice is usually made based on a biopsy obtained from the primary tumor. The main reason for choosing treatment based on a primary tumor tissue sample is that in general it is very difficult, and potentially dangerous for the patient, to obtain tissue from a metastatic tumor. Moreover, not all metastatic tumors are necessarily driven by the same pathway, given that the microenvironment of the metastatic tumor cells differs depending on tissue or organ location. Therefore a high priority question is how to obtain access to cancer cells that are representative for the various metastatic tumors in a patient? This is where the concept of circulating tumor cells joins the picture; capturing these rare cancer cells from blood may provide a potentially powerful approach to obtain a surrogate metastatic tissue sample, and to perform the necessary assays to identify the tumor-driving pathway, even on a single cell basis.

## 4. CTCs as a “Liquid Tumor Biopsy”

Capturing CTCs from blood has been named a “liquid biopsy”. CTCs which are released into the blood circulation by malignant tumors, both primary and metastatic cancer, can in principle be found in the patient’s blood, on the premise of a sufficiently sensitive and specific CTC capturing method. The latter still faces formidable challenges [22,23,24]. With currently available techniques CTCs can be captured from blood in extremely low numbers, from a few to a few hundred cancer cells per ml of blood. They hide among millions of white and billions of red blood cells and are detectable in only a minority of patients. CTC capture techniques in use are in general based on antibody-mediated binding of the epithelial membrane marker EpCAM on CTCs. By now it is generally accepted that probably many more CTCs are present in blood than can be captured this way, however they go undetected because these cancer cells probably exchanged epithelial for more stem cell like characteristics due to an epithelial mesenchymal transition process (EMT) [16,22]. These EpCAM-negative CTCs may prove to be a very important subset of CTCs, since they are thought to represent stem cell-like cancer cells responsible for metastasis, and do not respond to current therapeutic regimens. CTC-based diagnostics for therapy choice in patients with metastatic disease should include analysis of this subpopulation, which needs to be drug-targeted to enable long term disease control. Assuming that CTCs, both epithelial and mesenchymal type, derive from different metastatic sites, they are expected to be heterogeneous in many more aspects than only EpCAM expression, emphasizing the need for characterization of individual CTCs to optimize diagnostic value.

With time ideas about the clinical utility of CTC diagnostics have changed: from a prognostic test to an assay for prediction of therapy response enabling the right choice of (targeted) therapy in a patient with metastasis, and an assay to monitor the effect of the administered therapy. In contrast, the use of CTC counts as a screening or primary cancer diagnostic assay has not been clinically adopted [22]. This switch in clinical utility implicates that clinical trials for validating a CTC assay can become intrinsically shorter and with that, less expensive: clinical validation of an assay to predict therapy response may take a few months, instead of the five to ten years to validate a prognostic test.

## 5. Developments in CTC Capturing Technologies, the Past and the Future

Clearly, obtaining CTCs for diagnostics purposes is not a trivial task. The CTC field roughly exploded around a decade ago with the acquisition of Veridex by J&J and the subsequent FDA approval of the CellSearch assay, the first prognostic CTC assay developed by Leon Terstappen and clinically validated for patients with a variety of solid cancers, like metastatic breast, prostate and colon cancer [22]. An increasing number of engineering and biomedical/clinical groups started to explore the field, convinced that capture of CTCs was a technical challenge to be overcome by dedicated scientific work, with the promise to deliver a major step towards improved cancer diagnostics. However, different cancer types appeared to have their “own” type of CTCs, which can be very heterogeneous within one patient, and may be present either single or in a variety of “clusters” with leukocytes or platelets, and may even change phenotype in response to the blood environment. In many patients no CTCs were found at all. As a consequence, things turned out to be far more difficult than anticipated and it turned out impossible to capture CTCs with one standard device.

The considerable research effort to develop more efficient CTC capturing methods seems to stagnate currently: since our previous overview of 2011 [22], little essential progress has been made [25]. Although anti-body based capture methods, such as microfluidic approaches using geometrical features or magnetic beads coated with antibodies, have applied other antibodies than EpCAM, these developments are incremental an do not provide breakthroughs. Also, it has become clearer that selection based on physical parameters such as stiffness or electrical impedance have limitations in that they are applicable to specific cancers only [26,27]. All new methods proposed still have to prove their clinical value. Many novel technical approaches to capture CTCs emerged and were recently reviewed [28], for this reason we limit ourselves to a brief high level perspective of a few promising future directions towards capturing and analyzing both EpCAM positive and negative CTCs.

Since “positive” CTC selection (enrichment or purification) strategies introduce bias due to loss of specific CTC populations, a non-selective or minimally selective approach, only removing red blood cells, may turn out to be optimal, on the premise that literally all nucleated cells in the blood can be deposited on an appropriate surface substrate, which can be used to stain and rapidly scan the cells to identify CTCs and relevant CTC biomarkers.

Alternatively, “negative” selection approaches, meaning active removal of both red and white blood cells, should result in retrieval of most CTC populations. While red blood cell removal is relatively straightforward, e.g., by selective lysis, this is not the case for white blood cells. These are usually removed using CD45 antibodies which recognize all leukocyte subpopulations. Unfortunately it seems like some CTCs can also be CD45 positive, and such CTC populations will be lost for analysis [22]. Using instead suitable (combinations of) CD antibodies that specifically recognize leukocyte subpopulations may provide a potential solution for more specific removal of leukocytes. A non-answered question is why at least a subset of CTCs may be CD45 positive: one hypothesis is that CTCs may acquire CD45 while residing in the bone marrow stem cell niche.

Another drastically new approach which is currently being explored is based on extraction of CTCs from blood using a leukapheresis procedure, which is a way to enrich for white blood cells—the blood cell population which includes the CTCs. The circulating blood of the patient (around six liter) can be centrifuged or passed through external filters outside the body to separate and collect leukocytes—among which CTCs. A leukocyte-enriched leukapheresis sample can be used to obtain CTCs in various ways, among which a conventional CellSearch assay to capture EpCAM positive CTCs. Initial results indicate that using this approach CTCs can be found in many more patients [28,29]. However leukapheresis is not without risk to the patient, making it unlikely that this will easily become clinical routine in cancer patients. However, in cases where CTCs cannot be obtained in any other way (a one-time?) leukapheresis procedure may be an option. One might envision that, based on analysis of the captured CTCs, a CTC-specific biomarker can be identified e.g., by biomarker staining, sequencing, PCR, etcetera, for each individual patient, which can subsequently be used for standard blood sample-based capture during the further course of the disease in that specific patient.

After deposition of a CTC containing sample on a suitable surface, upcoming multiplex fluorescent staining technologies in combination with fast fluorescent digital scanners are expected to facilitate CTC recognition among many white blood cells [30]. Upon marking of the location of CTCs on the scanned surface by appropriate scanner software, CTCs may in principle be selected by the software, to subsequently perform sophisticated *in situ* molecular assays to measure the number of specific mRNA molecules and specific genomic mutations on a single cell basis [19,20,31,32,33]. In case of a large cell-covered surface area, microfluidics approaches can be developed to perform for example a PCR assay *in situ* within single drops generated locally on the surface e.g., by electro-wetting [34]. If a more complex assay like single cell DNA or transcriptome sequencing is needed, individual CTCs may be carefully removed for further analysis using a number of still exploratory techniques, like for example micro-manipulators, an optoelectronic trap [35,36,37] or a droplet-based technique [38,39].

## 6. Clinical Utility of CTC-Based Diagnostics in a Patient with Metastatic Disease: A Promise for the Future

### 6.1. Choosing the Right Drug Treatment

On the premise that the above-mentioned hurdles and issues will be solved, we expect that in the not too far away future from every cancer patient with metastatic cancer a blood sample will be taken to get hold of EpCAM positive and negative CTCs for companion diagnostics purposes, *i.e.*, *in situ* staining and counting of CTC subpopulations, and performing mRNA and DNA assays to identify the tumor driving signaling pathway together with specific causative mutations. Together this is expected to provide a picture of the heterogeneity of the CTC population and, inferred from that, of the metastatic tumors in the patient’s tissues and organs. Comparison with similar tests performed on primary tumor tissue (and metastatic tumor tissue if available) will provide additional information on heterogeneity and evolution of the tumor. Based on all information combined, the optimal targeted treatment can be chosen. Such a treatment may consist of more than one drug, for example depending on heterogeneity identified in CTC subpopulations. In case no, or an insufficient number of, CTCs can be captured by conventional blood sampling, the patient may be selected to undergo leukapheresis, potentially including identification of a “personal” CTC marker to subsequently facilitate repeated CTC capture from standard blood samples if needed.

### 6.2. Monitoring Treatment Response

Targeted drug treatment can be part of a more complex treatment schedule, for example including specific chemotherapy, local radiation treatment of metastatic locations, or in the future other systemic treatments which target tumor biology like synthetic lethality or immunotherapy [40,41]. What is urgently needed in any chosen treatment or treatment combination is a means to reliably quantify and monitor treatment response. At least with respect to targeted drug treatment, assessment of initial therapy response should be made as soon as possible to enable switching to another, more effective, targeted drug in case of primary resistance. This is on the premise of course that more targeted drugs will rapidly become available to choose from in this group of patients, either approved or off-label [42,43]. Current therapy response monitoring is routinely done using a variety of imaging modalities, e.g., CT scan, MRI, PET or combinations. Unfortunately these in general are not suited and lack sensitivity for early therapy response detection, for multiple reasons. The tumor response to targeted therapy is not necessarily associated with a measurable reduction in tumor size, and in addition may be hidden by an associated active inflammatory response [44]. For this reason alternative methods for therapy response assessment and monitoring of emerging resistance and tumor recurrence are being explored. Regular blood-based CTC counting has been shown to enable monitoring of therapy response on the premise that the patient’s blood contains a sufficient number of CTCs to perform reliable statistics to identify a reduction or increase in counts [45]. However CTC counts lack information on the signaling pathway that should be blocked by the chosen targeted therapy. Adding CTC-based assays for pathway activity, properly quantified and interpreted by appropriate software, are expected to enable monitoring of signal transduction pathway activity in response to targeted therapy and will provide valuable additional information on emerging drug resistance. To illustrate with an example, administration of tamoxifen in a breast cancer patient with an active estrogen receptor (ER) pathway would be expected to result in reduced CTC count, while associated CTC analysis for pathway activity should indicate that the ER pathway is not in active modus any more.

As already mentioned before, all this is not an option for the patient without a sufficient number of CTCs. For this reason and pushed by the rapidly developing sequencing field, another approach has been welcomed into the cancer diagnostics and monitoring field: cell free tumor DNA was shown to circulate in blood in sufficient amounts in metastasized patients, from which it can be relatively easily isolated and quantified by PCR or sequencing [46,47,48]. Algorithms comparing cell free DNA assay results in time enable comparison of the clinical status, *i.e.*, tumor load, of the patient on various sampling times. The lab of Bert Vogelstein at Johns Hopkins was the pioneer with development of a rather complex assay technology for quantification of tumor mutations in blood [46]. Next was Peter Campbell from Sanger/Wellcome trust who explored the approach to quantify tumor DNA rearrangements in blood, enabling a relatively simple quantitative PCR assay [47]. More recently Nitzan Rosenfeld and Carlos Caldas from Cancer Research UK described identification and quantification of cell free tumor DNA using advanced sequencing technologies [48]. They all provide clinical evidence that using quantitative measurements of tumor DNA in blood, it should be possible to monitor therapy response and recurrence in a variety of cancer patients. A disadvantage is that one or more patient-specific DNA mutations or rearrangements still need to be identified in tumor tissue using DNA sequencing technology, which then provide the basis for a blood-based monitoring assay. However the cell free DNA sequencing approach carries the promise to identify the monitoring biomarker directly from a blood sample [48].

### 6.3. CTCs or Cell Free DNA? Which Will It Be?

Cell free tumor DNA definitely has its merits for measuring therapy response, especially in cases where no CTCs are found, however it also has its drawbacks. In only a limited number of cancers the therapy choice-question can be answered by analysis of cell free circulating DNA, like demonstrated for certain cases of lung cancer driven by mutant EGFR [49]. For proper therapy choice in most patients with metastatic disease, intact cancer cell material needs to be available. With PCR or sequencing-based analysis of cell free DNA, mRNA, or non-coding RNAs in blood it will never be possible to establish which nucleic acid (and protein) components once existed (and interacted) together in an intact cancer cell—which is a prerequisite for analyzing the combined geno- and phenotype of the tumor cells—necessary for reliable companion diagnostics. In contrast, CTCs have the obvious advantage that their genotypic and phenotypic heterogeneity can be analyzed on an intact single cell basis.

## 7. Developments in the Pathology/Cytology Space: Towards Digital Pathology

The past decade a revolution in clinical pathology has been initiated through the development of fast digital scanner-software combinations which enable complete digitization of stained pathology and cytology slides, allowing the pathologist to zoom in on a tissue slide on his/her computer screen instead of under a conventional microscope [50,51,52,53]. Sophisticated algorithms to quantitatively interpret staining results will enable more objective pathology and cytology-based diagnostics. The first systems have recently obtained FDA approval for clinical use, opening up the space for many more applications. In the future, algorithms for such digital pathology systems can be developed to interpret and quantify CTC staining results. This is especially relevant for (high) multiplex fluorescent staining combinations necessary to recognize and biologically characterize CTC subtypes, since such staining results are too complex to reliably interpret manually. Where the CellSearch system still relies on handpicked counting of stained CTCs, such an implementation of digital pathology would mean a major breakthrough for CTC-based diagnostics. For the future we envision digital pathology platforms for staining-based CTC assays, including recognition and characterization with respect to signalling pathway activity, subtyping and counting, and enabling direct pathology comparison between CTCs and solid cancer tissue.

## 8. New *in Vitro* Human Cancer-on-a-Chip Models to Find out about CTC Behaviour and Characteristics

Reasons for the stagnation in the progress of CTC-based diagnostics include lack of evidence-based knowledge on: (1) molecular characteristics of CTC populations and associated migratory, intra-and extravasation, seeding, and metastatic growth behavior; (2) the effects of the blood and seeding site microenvironment on the metastatic growth potential of CTCs; (3) the importance of interactions between CTCs and other cell types encountered on the way to their organ destination, ranging from fibroblasts, endothelial cells, a variety of immune cells, to blood platelets and organ-specific cell types.

An exciting recent development combining (microfluidic) engineering technology, biophysics, biology, and clinical science to create a completely new type of human *in vitro* disease model systems, called “organ-on-chip” models, could help fill this knowledge gap on metastatic behavior of circulating tumor cells [54,55]. Such microfluidic systems can contain “micro-incubators” connected by micro-channels, allowing for 3D cell culture with controlled interaction between different cell types, and controlled fluid flow through the micro-incubators enabling for example continuous culture medium refreshment for prolonged culture and/or flow-through of immune cell populations to simulate *in vivo* blood flow. In addition to biochemical factors, specific *in vivo* biophysical conditions like hypoxia, increased pressure, and cell stretch can be mimicked. The “chips” can be microscope-slide sized and compatible with real time monitoring using optical techniques. The application of this technology to develop “cancer-on-a-chip” models will enable detailed investigations of the process of cancer metastasis, including studies on behavior and molecular characteristics of tumor-specific circulating tumor cells [56].

## 9. Conclusions

Challenges to progress towards clinically approved CTC-based diagnostics are multifold, but the reward in terms of quality of patient care, especially the patient with metastatic disease, will be worth the effort.

Technological requirements for a device to capture and handle CTCs from a blood sample may differ depending on tumor type, and are dependent on the ultimate goal, that is enumeration and/or molecular characterization. Especially development of novel techniques to capture EpCAM negative CTCs is an ongoing challenge but high priority. To improve recognition and capture of EpCAM negative CTCs from blood it will be necessary to study cancer cells and their characteristics during the metastatic process, which will be possible using organ-on-chip cancer model systems.

Capturing technologies should enable integration of sample processing steps for subsequent single CTC diagnostic assays like multiplex fluorescent staining and nucleic acid analysis. When captured cells are deposited on a surface like a glass slide to be stained and characterized, algorithms should be developed for automated data interpretation. Ultimately, clinical interpretation will require complex software to deal with both multiplex fluorescent biomarker stainings and DNA/RNA assay results from a potentially highly heterogeneous population of CTCs, and compare results at different time points. Defining clinical utility and enabling clinical decisions based on CTC analysis and finally obtaining regulatory clearance requires rigorous clinical trials.

Excellent and creative multidisciplinary research will be needed to push the CTC field forward towards the clinic, bringing together physicists, engineers, cancer biologists and clinicians/oncologists. On that premise CTC diagnostics stands at the beginning of a very promising future, and if successful, will create a breakthrough in cancer treatment!
